# Characterization of the complete chloroplast genome of *Actinidia hemsleyana*

**DOI:** 10.1080/23802359.2021.1993100

**Published:** 2021-10-20

**Authors:** Qi Xiaoqiong, Xie Xiaodong, Yang Mingjuan, Arif Atak, Zhong Caihong, Li Dawei

**Affiliations:** a Hubei Key Laboratory of Purification and Application of Plant Anti-cancer Active Ingredients, School of Chemistry and Life Science, Hubei University of Education; b CAS Engineering Laboratory for Kiwifruit Industrial Technology, Wuhan Botanical Garden, Chinese Academy of Sciences, Wuhan, China; c School of Biological and Environmental Engineering, Xi'an University, Xi'an, China; d Department of Viticulture, Ataturk Horticultural Central Research Institute, Yalova, Turkey; e Center of Economic Botany, Core Botanical Gardens, Chinese Academy of Sciences, Wuhan, China

**Keywords:** *Actinidia hemsleyana*, chloroplast, endanger species, phylogenetic analysis

## Abstract

The completed chloroplast genome sequence of *Actinidia hemsleyana*, collected from eastern China, was firstly determined using next-generation sequencing (NGS) and bioinformatic analysis. The length of cp genome was 156,845 bp, including a large single-copy (LSC) region of 88,666 bp and a small single-copy (SSC) region of 20,543 bp, which were separated by a pair of inverted repeats (IRs) of 23,818 bp. The cp genome contained 132 genes, including 83 protein-coding genes, 41 tRNA genes, and 8 rRNA genes. The overall GC content was 37.19%, whereas the corresponding values of the LSC, SSC, and IR regions were 35.46%, 31.09%, and 43.06%, respectively. Phylogenetic analysis based on whole cp genome sequences of 20 *Actinidia* species showed that *A. hemsleyana* has the closest relationship with *A.latifolia*.

*Actinidia hemsleyana* is one of the endangered *Actinidia* species in China (Huang 2014). The plant has rusty-brown strigose hairs on leaves and floral branchlets. The fruit is small (4–5 g), with green-fleshed and a light acid flavor. The plant grows vigorously and shows good resistance to abiotic stress, which provides potential materials for the improvement of kiwifruit in the future breeding programs. According to the morphological characteristics, *A. hemsleyana* was considered to be closer to *A. callosa* and *A. henryi*. However, its phylogenies was more close to *A.rufa* (Chat et al. 2004) basing on AFLP markers (Li 2006). Considering that cp genome played an important role in revealing the origin of the evolution and phylogeny of *A. hemsleyana*, its complete cp genome was assembled and phylogenic position was analyzed in this paper.

Leaf samples of *A. hemsleyana* were obtained from Wuyanling National Nature Reserve, Zhejiang Province (27°28'N, 119°45’E). A specimen was deposited at the Herbarium of National *Actinidia* Germplasm Repository of China, Wuhan Botanical Garden, the Chinese Academy of Sciences (http://english.wbg.cas.cn/; LI Dawei; lidawei@wbgcas.cn) under the voucher number Acs10006. Total genomic DNA was extracted from fresh leaves using a DNeasy Plant Mini kit (QIAGEN, Hilden, Germany). It was sequenced on Illumina Hiseq 2000 platform (Illumina, San Diego, CA). With the chloroplast genome of *A. chinensis* as the reference (Yao et al. 2015), the complete chloroplast genome was assembled using the clean reads by the NOVOPlasty (Dierckxsens et al. 2017). The annotation of cp genome was accomplished by Geneious 11.0.4 software (Biomatters Limited, NZ). Finally, the complete cp genome sequence was submitted to the GenBank with accession number "MT740251.1" and "Characterization of the complete chloroplast genome of *Actinidia hemsleyana*" title

The complete cp genome sequence of *A. hemsleyana* was 156,845 bp in length and consists of a large (LSC, 88,666 bp) and a small (SSC, 20,543 bp) single-copy regions, separated by a pair of identical inverted repeats (IR, 23,818 bp). The cp genome encoded 132 genes, of which 114 were unique genes (80 protein-coding genes, 30 tRNAs, and 4 rRNAs). The overall GC content was 37.19%, whereas the corresponding values of the LSC, SSC, and IR regions were 35.46%, 31.09%, and 43.06%, respectively.

To confirm the phylogeny of *A. hemsleyana*, a molecular phylogenetic tree was constructed based on the whole cp genome sequence of other 19 *Actinidia* species, with *Clematoclethra scandens* subsp. *hemsleyi* (KX345299.1) as outgroup. The Maximum-Likelihood (ML) tree was constructed using MEGA 6.0 with 1000 bootstrap replicates (Swofford 2003). The results showed that *A. hemsleyana* was closely related to *A.latifolia* (Figure 1).

**Figure 1.**
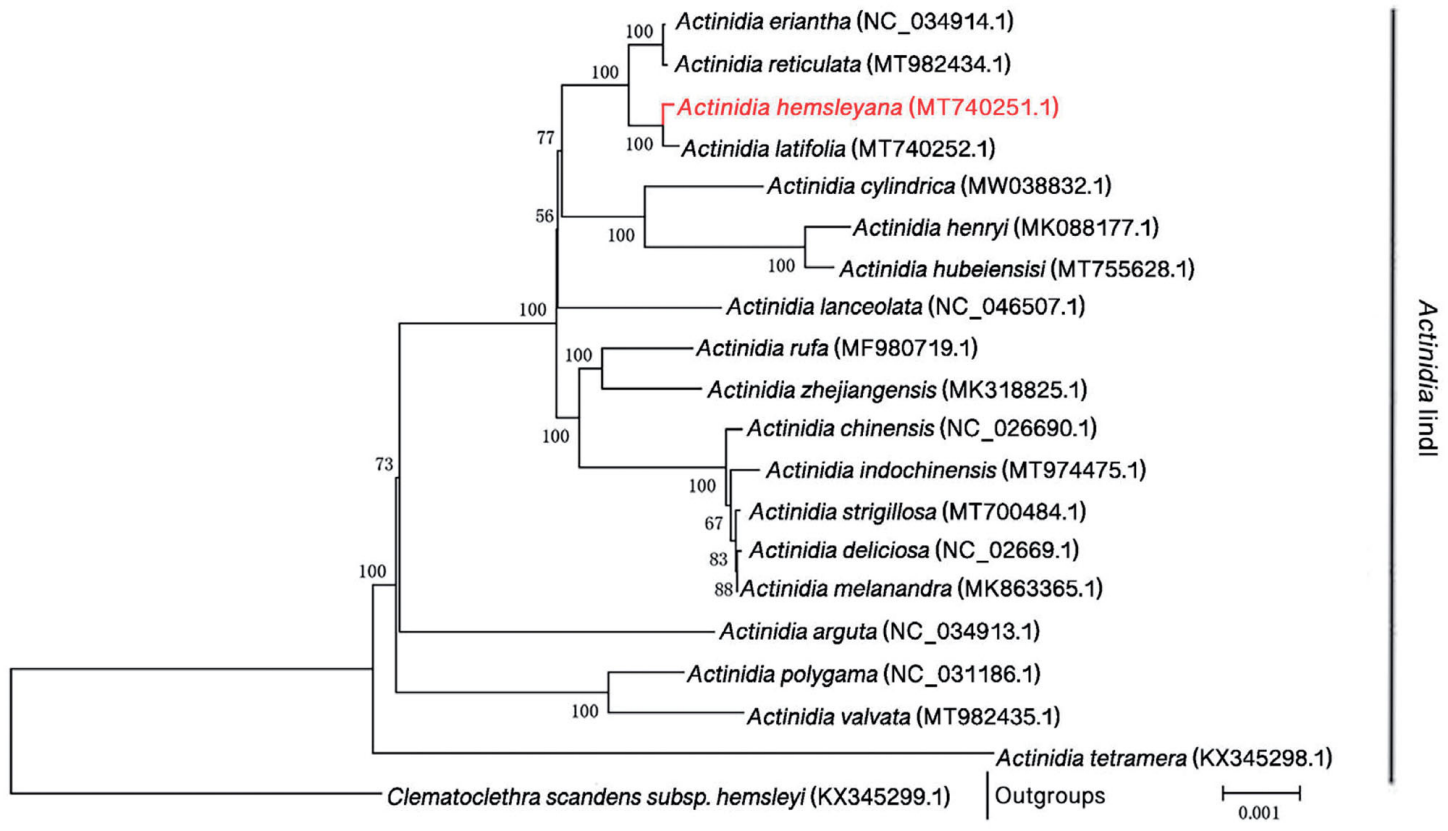
Phylogenetic position of *A. hemsleyana* in genus *Actinidia* as inferred by MP analyses of chloroplast genome sequences. Numbers above the lines indicate the maximum likelihood bootstrap value >50% for each clade.

## Data Availability

The genome sequence data that support the findings of this study are openly available in GenBank of NCBI at (https://www.ncbi.nlm.nih.gov/) under the accession no. MT740251.1. The associated BioProject, SRA and Bio-Sample numbers are PRJNA743141, SRR15018373 and SAMN19998353, respectively.
